# Late Pneumolabyrinth May Be Induced by Old Penetrating Injury: Possibility of Undiagnosed Posttraumatic Perilymphatic Fistula

**DOI:** 10.1155/2015/506484

**Published:** 2015-03-26

**Authors:** Takahiro Nakashima, Keiji Matsuda, Takumi Okuda, Tetsuya Tono, Minoru Takaki, Tamon Hayashi, Yutaka Hanamure

**Affiliations:** ^1^Department of Otorhinolaryngology, Head and Neck Surgery, Faculty of Medicine, University of Miyazaki, 5200 Kihara, Kiyotakecho, Miyazaki 889-1692, Japan; ^2^Department of Otorhinolaryngology, Head and Neck Surgery, Kagoshima City Hospital, No. 20-17, Kajiyacho, Kagoshima 892-8580, Japan

## Abstract

Traumatic pneumolabyrinth is a relatively rare entity. We report the case of a unilaterally deaf woman with pneumolabyrinth who had suffered penetrating injury 15 years ago. This past history indicated that the case was late pneumolabyrinth occurring from undiagnosed old posttraumatic perilymphatic fistula. In Japan, most cases of traumatic pneumolabyrinth are caused by penetrating injury with an ear pick. Dizziness often improves within several months. Immediate surgical intervention is recommended for hearing loss, but the hearing outcome is not satisfactory. An appropriate strategy should be selected based on the interval to surgery, bone conduction hearing level at disease onset, stapes lesions, and location of air.

## 1. Introduction

Pneumolabyrinth is a condition caused by air in the labyrinth, which indicates that a perilymphatic fistula (PLF) is present in the affected ear. However, patients with traumatic PLF seldom develop pneumolabyrinth. Here, we report a case of pneumolabyrinth that may have been caused by a penetrating injury of the tympanic membrane that occurred 15 years before presentation. This interesting case suggests that pneumolabyrinth may be among the differential diagnoses for recurrent dizziness.

## 2. Case Report

A 25-year-old woman was admitted to hospital with a complaint of longstanding right-sided profound hearing loss. Her hearing impairment had begun 15 years ago. At that time, she suffered traumatic tympanic membrane perforation due to penetration by an ear pick. She had sudden profound hearing loss at the onset of injury and it was unclear that she had felt vertigo after injury. Conservative therapy was performed at ENT clinic nearby. Although perforation of tympanic membrane was healed completely, profound hearing loss had remained. She mentioned the degree of hearing loss had not changed. She had felt lightheadedness about 7 years ago.

**Figure 1:**
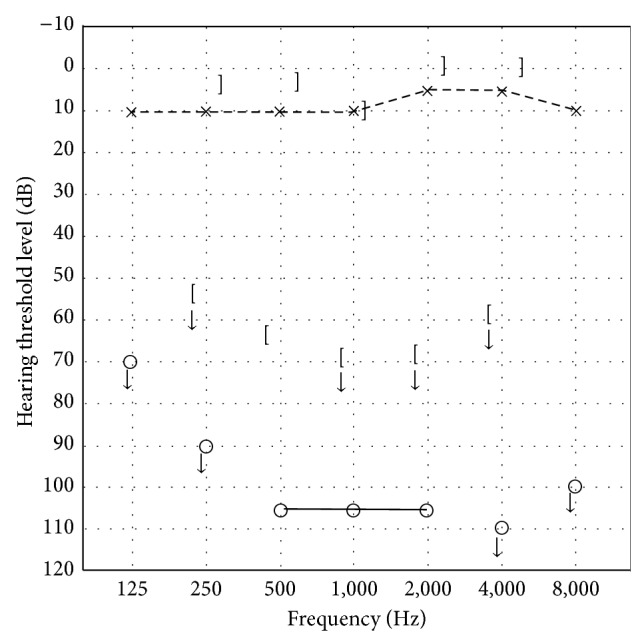
Audiogram on hospital on day 1. The right ear showed profound sensorineural hearing loss.

**Figure 2:**
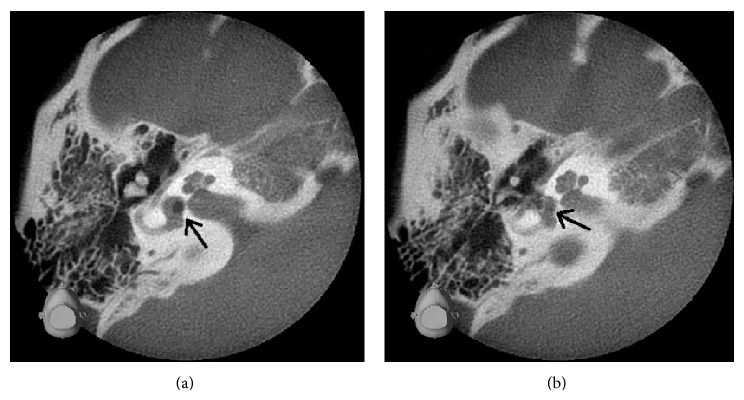
Axial views of the right ear on cone-beam computed tomography on hospital on day 1, showing a round low density area (a) and intrusion of stapes (b) (black arrows).

An otoscopic examination revealed no perforation in her right ear, but pure tone audiometry showed right profound hearing loss (Figure 1). Imaging of temporal bone using cone-beam computed tomography (CBCT) showed a round low density area in the vestibule and intrusion of stapes into the vestibule (Figure 2). A diagnosis of pneumolabyrinth was made and PLF was suspected. The patient refused surgical intervention and chose to receive outpatient follow-up and no medication.

**Figure 3:**
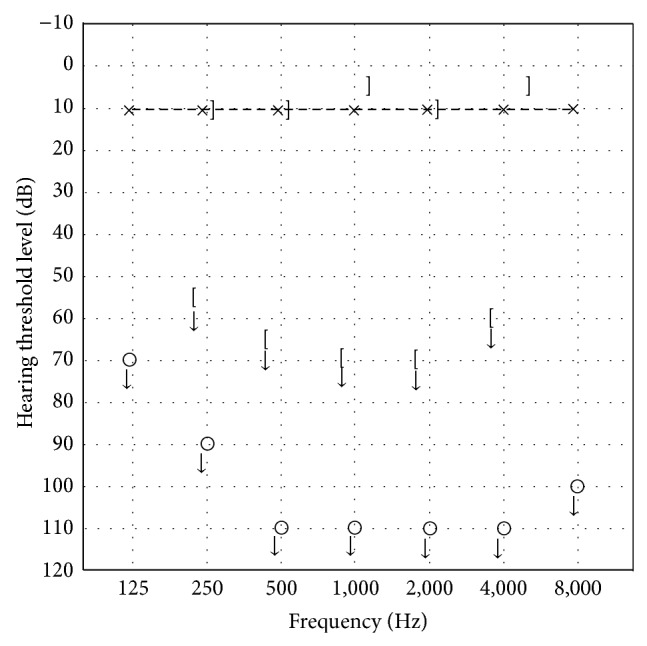
Follow-up audiogram performed 2 years later. Profound sensorineural hearing loss in her right ear had not changed.

**Figure 4:**
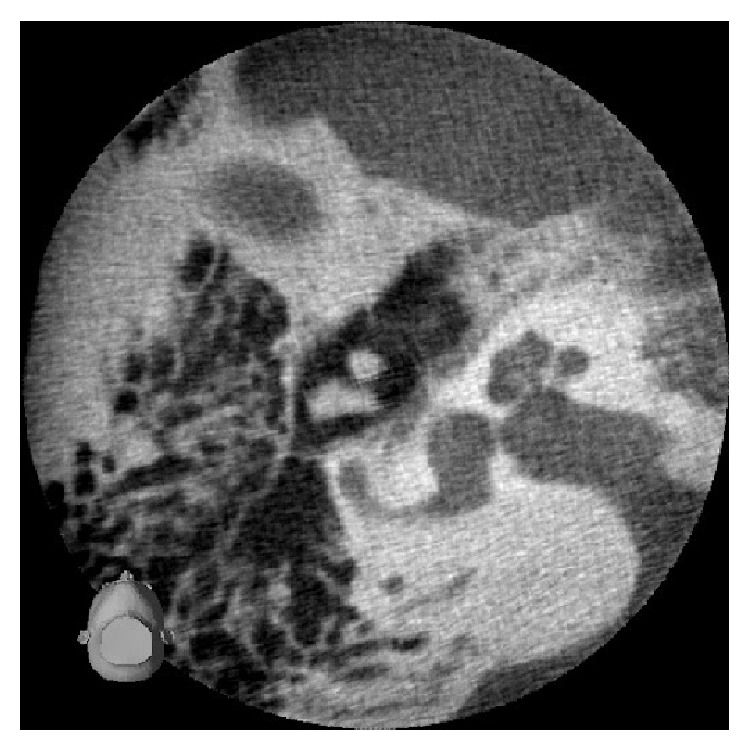
Axial view of the right ear on cone-beam computed tomography obtained 2 years after initial presentation. The air bubble apparent in Figure 2(a) had disappeared.

Two years later, she was admitted again for follow-up of hearing loss. She sometimes had dizziness or lightheadedness. Pure tone audiometry showed right profound hearing loss similar to before (Figure 3). Equilibrium tests showed no positional or positioning vertigo. A caloric test indicated that right vestibular function was slightly decreased, but not abolished. Reexamination by CBCT showed that air that had been present in the right vestibule had disappeared (Figure 4). We recommended probe tympanotomy to confirm and cure PLF, but the patient rejected surgical intervention and requested course observation.

## 3. Discussion

The number of reports on pneumolabyrinth is smaller than those on PLF but has shown a slight increase due to improved accuracy of imaging modalities. Hidaka et al. [1] suggested that about 40% of cases of pneumolabyrinth are caused by blunt trauma to the head, followed by penetrating injury. In Japan, however, penetrating injury is the most common etiology because of a habit of cleaning the external ear canal using an ear pick.

According to medical interview, profound hearing loss occurred immediately after injury and was supposed to be permanent. Intrusion of stapes found in CBCT image indicated the possibility of old posttraumatic PLF. Symptoms associated with PLF tend to appear rapidly and the degree of hearing loss varies from mild to profound. Subacute deterioration of hearing level has been documented in several reports [2–6] and all cases showed progression of hearing loss within 2 weeks. One case had an apparent causative event and all received conservative therapy.

The unique aspect of this case is the long interval between injury and diagnosis of pneumolabyrinth by CBCT. It was impossible to make sure that pneumolabyrinth had occurred soon after injury. Intrusion of stapes suggested that there had been posttraumatic PLF. There was no causative event other than penetrating injury by ear pick 15 years ago. Air that enters the inner ear usually disappears within several weeks. Pneumolabyrinth detected by CBCT suggested that air was trapped recently and that PLF did not heal completely. In the patient with PLF, elevated atmospheric pressure in the middle ear cavity may induce fainting and dizziness. If excessive elevation of the middle ear pressure happens, entrapment of air in the vestibule may occur. Nurre et al. [7] described a case of traumatic pneumolabyrinth due to a temporal bone fracture that happened 2 years earlier. Pneumolabyrinth may be a clue to find undiagnosed PLF that is among the differential diagnoses for dizziness.

Treatment of dizziness has a good prognosis, but treatment of hearing loss is difficult and often unsuccessful. Most reports have shown unsatisfactory hearing outcomes regardless of the treatment strategy. Hidaka et al. [1] suggested that there is no difference in efficacy between conservative therapy and surgical therapy. Tsubota et al. [5] proposed three predictive factors for hearing improvement: the interval until surgery, bone conduction hearing level at onset of disease, and stapes lesions. Probe tympanotomy should be performed as soon as possible and several authors have recommended surgical intervention within 2 weeks from disease onset.

Tsubota et al. [5] found that a bone conduction level (BCL) of >65 dB HL at onset of disease resulted in a poor hearing outcome. Hidaka et al. [1] found no BCL deterioration over 10 dB in patients with a preoperative BCL >65 dB HL, but a rate of worsening of 29% in those with BCL <65 dB HL preoperatively; however, this may reflect differences in the selected cases. Tsubota et al. [5] described 15 cases up to 2009 and Hidaka et al. [1] added another 16 cases reported since 2009. The earlier 15 cases had worse BCL and more of the cases were treated surgically. Regarding stapes lesions, Sarac et al. [8] pointed out that cases with no or mild stapes intrusion had good hearing outcomes after surgery, whereas those with deeply intruded stapes may not have improved hearing after surgery and may suffer from additional inner ear damage due to surgery. It was also concluded that stapes surgery should be performed early to avoid a late complication of inner ear dysfunction due to fibrosis in the labyrinth.

Hidaka et al. [1] also found that the location of air in the labyrinth is an important prognostic factor. In cases with air in the cochlea, a good hearing outcome can be expected, whereas hearing improvement may not always be achieved in cases with air trapped in the vestibule. Poor hearing results are also likely if air is present in both the cochlea and the vestibule. These clinical results are supported by findings of Kobayashi et al. [9, 10] using measurements of endocochlear DC potential, cochlear microphonic potential, and summating potential in guinea pig under conditions of air in the scala vestibuli and scala tympani. All potentials decreased temporarily and recovered with air in the scala tympani. In contrast, all potentials decreased without recovery with air in the scala vestibuli. In histological analysis, structures such as the organ of Corti, Reissner's membrane, spiral ganglion, and stria vascularis retained normal morphology in animals with air in the scala tympani, whereas collapse of the Reissner's membrane was observed in those with air in the scala vestibuli. Based on these results, it was suggested that air entrapped from an oval window might cause greater inner ear damage than that from a round window in pneumolabyrinth. These findings provide useful information on the pathogenesis of pneumolabyrinth.

Prevention from worsening of hearing loss is important. Achache et al. [11] emphasize that labyrinthine concussion should be systematically suspected in cranial trauma. Penetrating injury by an ear pick is sometimes accompanied with dizziness. If there is mixed or sensorineural hearing loss, careful follow-up to check hearing status is mandatory. Additionally, thin-sliced computed tomography of the temporal bone should be performed to examine the condition of stapes and inner ear.

## 4. Conclusion

Pneumolabyrinth may occur in case PLF is present, even if the interval between diagnosis and causative event is very long. An appropriate decision on the treatment strategy requires consideration of the timing of surgery, the bone conduction level at onset of disease, stapes lesions, and location of air in the inner ear.
